# Prevalence of frailty among community dwelling older adults in receipt of low level home support: a cross-sectional analysis of the North Dublin Cohort

**DOI:** 10.1186/s12877-017-0508-2

**Published:** 2017-06-07

**Authors:** Sara Kelly, Irene O’Brien, Karla Smuts, Maria O’Sullivan, Austin Warters

**Affiliations:** 1 North Dublin Home Care Ltd., 2 Malahide Road, Fairview, Dublin 3, Ireland; 2 Clinical Medicine, Trinity Centre for Health Sciences, St James’s Hospital, Dublin 8, Ireland; 3 Health Service Executive Healthcare Facility, Ballymun, Dublin 9, Ireland

**Keywords:** Frailty, Home help, Domiciliary care, Ageing, Clinical frailty scale

## Abstract

**Background:**

There is increasing demand for formal government funded home help services to support community-dwelling older people in Ireland, yet limited information exists on the health profiles of this group, especially regarding frailty. Our aim was to profile a large cohort of adults in receipt of low level home help and to determine the prevalence of frailty.

**Methods:**

A total 1312 older adults, (≥ 65 years) in receipt of low level home help (< 5 h per week) were reviewed by community nurses and frailty was assessed using the Clinical Frailty Scale (CFS) in this cross-sectional study. Characteristics of the group were compared between males and females and prevalence of frailty was reported according to gender and principal care. Associations between frailty and a number of variables were explored using bivariate and regression analysis.

**Results:**

The cohort of low level home-help users was a mean age of 82.1 (SD 7.3) years, predominantly female (70.6%) and over half (69.2%) lived alone. The prevalence of frailty in this population was 41.5%, with subjects primarily considered mildly (23.2%) or moderately frail (14.5%) by the CFS. A further 38.4% were classed as vulnerable. The degree of frailty did not differ significantly across the younger categories aged 65–84 years. However, in the oldest age groups, namely 90–94 and >95 years, moderate frailty was significantly higher relative to the younger groups (21% and 34%, *p* < 0.05, *p* < 0.01 respectively). Home help hours significantly correlated with frailty (rs = 0.371, *p* < 0.001) and functional dependency (rs = 0.609, *p* < 0.001), but only weakly with age (rs = 0.101, *p* = 0.034). Based on regression analysis, determinants of frailty included greater dependency (Barthel score), higher home help hours, non-self-caring and communication difficulty, all of which significantly contributed to the model, with a r squared value of 0.508.

**Conclusion:**

A high prevalence of frailty (41.5%) was documented in this population which associated with higher home help utilisation. Frailty was associated with greater functional dependency, but not strongly with chronological age, until after 90 years. These findings highlight opportunities for developing intervention strategies targeted at ageing in place among home help users.

**Electronic supplementary material:**

The online version of this article (doi:10.1186/s12877-017-0508-2) contains supplementary material, which is available to authorized users.

## Background

### Background to the study

In 2015, 8.5% of the world’s population was aged 65 and over and expected to reach 1.6 billion by 2050, representing 16.7% of the World’s total population [1]. Irish projections mirror this trend, with adults aged 65 years and over predicted to rise from 532,000 in 2011 to an estimated 1.4 million by 2046 [2]. While this increase in longevity is a success of our times, inevitably it has implications for the planning and delivery of health and social care. In this regard, ‘ageing in place’ and the promotion of independent living is central to current government health strategies [3, 4]. The trajectory from independent living to supported care is associated with functional decline and frailty, common features in community dwelling older adults [5–7] that increase the risk of adverse outcomes [8, 9]. The majority of older people over 65 years in Ireland live at home [10] supported by varying degrees of informal and/or formal community based medical, social and other care, including formal home help. The latter also termed domiciliary care in other regions.

### Aims

There are limited published data on the profile of the home help population regarding determinants of dependency, hence the present study focused on this cohort, its characteristics and degree of frailty. Based on a large sample of community dwelling older adults in receipt of low level home support, this study aimed to document the characteristics of the group, and to determine the prevalence of frailty and its associated factors amongst this cohort.

### Summary of existing literature

Community dwelling older adults who interface regularly with health care services at home, such as home help, represent a potentially interesting group for intervention strategies targeted at maintaining independence, for example physical activity or connected health programmes [11]. An estimated 8.2% of community dwelling Irish older adults aged 65 and over, utilise formal home help according to data from the Irish Longitudinal study of Aging (TILDA) [12]. The utilisation of these formal home help services increases gradually with age, from 1.6% of adults aged 65–69 up to 30.3% of those 85 years and older [12]. Access to formal government funded home help services varies across countries; in Ireland, provision is determined by an assessment of need conducted by a health professional and is not at present income assessed. A number of factors are taken into account, including a person’s clinical condition, cognitive status, dependency level, and current informal home help available to them. If a person is deemed to be in need of formal home care, they will either be funded for generic home help hours (<5 h/week) or a complete home care package (>5 h/week) by the Irish Health Service Executive. Home Care Packages are frequently reviewed, with revisions often leading to an increase in home help hours and costs. Highly dependent older persons in the community who have limited informal support are provided with intensive home care packages (IHCPs), however due to scarcity of resources and cutbacks in social care budget experienced in recent years, the allocation of IHCPs are highly restricted.

Frailty is an age-related, progressive condition, characterised by extreme vulnerability which exposes individuals to adverse health related outcomes such as falls, reduced mobility and independence, with increased hospitalisation, disability, cognitive decline and mortality [13–18]. While there is little consensus on operationalising the concept, most measures of frailty are on a continuum from non-frail, pre-frail to frail, representing a transition phase between successful aging and disability [13, 19]. A number of different approaches are currently used to identify frailty in the elderly. This includes the objective phenotypic approach [20], based on the presence of a number of adverse physical factors, and the cumulative deficit approach which measures the total number of health deficits of a given person, namely the Frailty Index [21]. Approaches based on clinical judgement have also recently been developed [22]. Frailty is considered a marker of biological ageing and a better predictor of functional decline, health care use and mortality [21, 23, 24] than chronological ageing alone. Moreover, evidence suggests that frailty indicators in community-dwelling older people may be useful in identifying people who could benefit from disability prevention [25] or other intervention programs.

Prevalence estimates of frailty among older adults in the community vary considerably, in part, due to methodological differences in frailty definitions and assessment tools applied as well as variations in the populations studied. A systematic review based on 21 studies with 61,500 participants, reported that 41.6% of community dwelling older adults were pre-frail with a further 10.7% considered as frail [14]. Frailty was more prevalent in women (9.6%) than men (5.2%) and increased steadily with age ranging from 4% in those aged 65–69 years up to 26% in persons over 85 years [14]. Further studies have determined 23.5–42% prevalence rates of frailty amongst community dwelling Irish older adults [24, 26].

### Contribution to the field

To our knowledge, there is limited information available on frailty rates amongst those utilising state-funded home support. We propose that better understanding the profile of older populations receiving in-home support, specifically low-level home help, may have applications for health service planning, and in identifying opportunities for other in-home strategies that promote and enable ageing in place. Placing our focus on older people receiving low-level home help (<5 h/week) will give insight into the characteristics of those who require only minimal input from formal home care services. Elucidating determinants of frailty in this particular cohort may generate potential opportunities for frailty prevention strategies and thus limit the need for future increases in home help hours for those receiving low level, less costly home support.

## Methods

### Study sample and population

A cross-sectional analysis was carried out to profile a cohort of low level home help users in Dublin and to determine prevalence of frailty. Data were extracted from the Dublin North City and Dublin North West Health Service Executive (HSE) administrative area databases. This administrative area has a population of 34,240 aged 65 years and over [27, 28]. Participants were identified based on the following inclusion criteria: older adults aged 65 years and over, living at home in the selected administrative area and receiving formal low level home help, defined as ≤5 home help hours per week, from the HSE. In Ireland, state-funded home help encompasses personal care and domestic help, is allocated based on a needs assessment by a health care professional, and is not presently income assessed. Those receiving in excess of 5 h home support per week are formally regarded as receiving enhanced home care (i.e. home care packages) and were excluded from the study.

Once service users were identified, an in-home review was scheduled and conducted with each older person during the period of January 2014 to April 2015. Reviews were performed by community nurses using a routine assessment form, namely the Common Summary Assessment Report (CSAR) [29].A total of ten public health nurses (PHNs) carried out assessments on all study participants. All nurses were highly experienced in carrying out assessments using the CSAR methodology. In addition, an assessment of frailty was added to this review, which was not in routine use. Written informed consent was obtained from all participants to complete CSAR assessments. All data were anonymised before use. Analysis and evaluation of data was approved by the HSE, Community Health Organisation Area 9, Dublin and by the Research Ethics Committee in Dublin City University, Dublin (DCUREC/2015/236).

### Demographic and background information

Information on age, gender, marital status, living status, principal carer and communication ability was derived from the CSAR form. Ability to communicate was rated using a routine clinical score in the CSAR, from 1 to 5 as follows: no problems, retains most information and can indicate needs verbally, difficulty speaking but retains information and indicates needs non-verbally, can speak but cannot indicate needs or retain information, no effective means of communication. The number of hours of home help allocated to the service user was documented, ranging from 30 min to a maximum of 5 h per week. Insufficient information was available from the routine data to determine factors such as socioeconomic status, health and cognition status or other detailed health or lifestyle parameters.

### Assessment of frailty and independence

Frailty was assessed using the Clinical Frailty Scale (CFS) which provides summary measures of the level of frailty between 1 (very fit) and 9 (Terminally ill) and has been validated in clinical settings [22]. Independence was assessed using the modified Barthel Index [30] which evaluates feeding, bathing, grooming, dressing, bladder control, toileting, chair/bed transfer, mobility and stair climbing. Subjects were classified by a score ranging from 0 (complete dependence) to 20 (complete independence).

### Statistical analysis

Statistical analysis was performed using IBM SPSS (Version 22.0). Descriptive statistics were used to characterise the cohort and presented as means and standard deviations, and comparisons were drawn between males and females. Comparisons between groups were conducted using one way analysis of variance for continuous data and cross tab with chi-squared tests for categorical variables. The Tukey HSD test was used as a post-hoc analysis to explore differences in mean CFS scores between age categories and mean home help hours between frailty categories. Spearman’s rank order coefficient (rho) was used to measure associations between clinical frailty and a number of factors including age and home help hours. Finally, multiple regression analysis was performed to identify factors associated with CFS score. All statistical tests were two-tailed and a 5% significance level was maintained.

## Results

### Characteristics of the study population

A total of 1312 urban, community dwelling older adults, (≥ 65 years) in receipt of low level home help (mean 2.27, SD 1.32 h per week) were identified and reviewed in the study time period, representing 3.83% of older persons in the defined administrative area.

Characteristics of the cohort are displayed in Table 1 (end of manuscript). The mean age was 82.1 (SD 7.3) years, with the majority (84%, *n* = 1099) aged between 75 and 94 years. In line with ageing trends, there was a female preponderance (71%) with women being significantly older than men (mean 83, SD 7.7 v 80, SD 7.1 years respectively) and more likely to be classed as widowed or single (*p* < 0.001). The study population largely lived alone (69%, *n* = 887), were self-caring (86%, *n* = 1119), over half were widowed (54%, *n* = 691) and most had no documented communication difficulties (83%, *n* = 1088). The mean modified Barthel score was 17.44 (SD 2.86), with participants most frequently classed as low dependency (47.6%, *n* = 624) in the areas of feeding, bathing, grooming, dressing, bladder control, toileting, chair/bed transfer, mobility and stair climbing (Table 1).

**Table 1:** Characteristics of community-dwelling older adults receiving low level home-support, overall and by gender (*n* = 1312)

Characteristic	Overall *N* = 1312	Male	Female	*P* value
Gender, *n* (%)	-	386 (29.4)	926 (70.6)	<0.001
Age in years, mean (SD)	82 (7.3)	80 (7.1)	83 (7.7)	<0.001
Age category (y), *n* (%)				
65–69 years	94 (7.2)	44 (11.4)	50 (5.4)	<0.001
70–74 years	116 (8.9)	48 (12.4)	68 (7.3)	0.003
75–79 years	224 (17.1)	71 (18.4)	153 (16.5)	0.426
80–84 years	346 (26.4)	103 (26.7)	243 (26.2)	0.894
85–89 years	314 (24)	69 (17.9)	245 (26.5)	0.001
90–94 years	180 (13.8)	45 (11.7)	135 (14.6)	0.155
≥ 95 years	35 (2.7)	6 (1.6)	29 (3.1)	0.104
Living status, *n* (%)				
Lives alone	887 (69.2)	265 (68.7)	622 (67.2)	0.581
With others	395 (30.8)	112 (29.0)	283 (30.5)	0.581
Marital status, *n* (%)				
Married	266 (20.8)	101 (26.2)	165 (17.8)	<0.001
Widowed	691(53.9)	139 (36)	552 (59.6)	<0.001
Single	261(20.4)	105 (27.2)	156 (16.8)	<0.001
Separated/Divorced	63 (4.9)	36 (9.3)	27 (2.9)	<0.001
Principal carer, *n* (%)				
Self-caring	1119 (85.9)	320 (82.9)	799 (86.3)	0.146
Spouse/Partner	75 (5.8)	39 (15.3)	36 (3.9)	<0.001
Family	108 (8.3)	23 (6.0)	85 (9.2)	0.055
Home help hours, mean (SD)	2.27 (1.3)	2.28 (1.3)	2.24 (1.3)	0.627
Communication difficulties, *n* (%)				
Absent	1088 (83.1)	308 (79.8)	780 (84.2)	0.057
Present	222 (16.9)	77 (20.0)	145 (15.7)	0.057
Barthel Index Score^a^, mean (SD)	17.44 (2.9)	17.76 (3.0)	17.31 (2.8)	<0.001
Barthel Scale^b^, *n* (%)				
Independent	415 (31.7)	158 (40.9)	257 (27.8)	<0.001
Low Dependency	624 (47.6)	161 (41.7)	463 (50.0)	0.008
Medium Dependency	224 (17.1)	50 (13.0)	174 (18.8)	0.012
High Dependency	44 (3.4)	15 (3.9)	29 (3.1)	0.479
Maximum Dependency	3 (0.2)	0	3 (0.3)	0.264

^a^Barthel Index Score: ranges from 1 to 20 with lower scores indicating dependency

^b^Barthel Scale categories: independent (score of 20), low dependency (score of 16–19), medium dependency (score of 11–15), high dependency (score of 6–10), maximum dependency (score of 0–5)

### Prevalence of Frailty

**Fig. 1:**
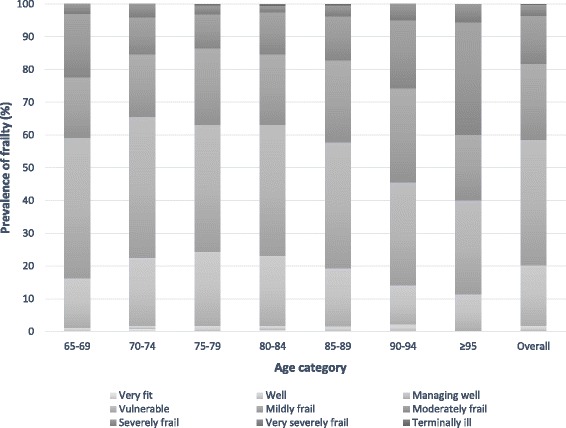
Prevalence of frailty categories (CFS) according to age category and overall in community-dwelling older adult low level home-help users (*n* = 1312)

The prevalence of frailty in older adults receiving low level home help was 41.5% (*n* = 540) based on CFS 5–9 (Table 2, Fig. 1), comprised primarily of mildly (23.2%, *n* = 302) or moderately frail (14.5%, *n* = 190) individuals. Of note, a further 38.4% (*n* = 499) of the cohort were classed as vulnerable i.e. CFS 4. Few were severely frail, very severely frail or terminally ill as may be expected among older people receiving low level home help [3.2% (*n* = 42), 0.01% (*n* = 1) and 0.3% (*n* = 4) respectively; CFS 7–9]. Equally, at the other extreme, a low number were very fit (0.2%, *n* = 3) or well (1.5%, *n* = 19) corresponding with CFS 1 and 2 (Table 2, Fig. 1).

**Table 2:** Prevalence of frailty overall, according to gender (*n* = 1302)

Clinical Frailty Scale, *n* (%)	Overall	Gender		
		Female	Male	*p*-value*
Very Fit - Managing Well (CFS 1–3)	263 (20.2)	173 (18.8)	90 (23.4)	0.060
Vulnerable (CFS 4)	499 (38.3)	347 (37.8)	152 (39.6)	0.110
Mildly Frail (CFS 5)	302 (23.2)	225 (24.5)	77 (20.1)	0.082
Moderately Frail (CFS 6)	190 (14.6)	144 (15.7)	46 (12.0)	0.084
Severely Frail- Terminally ill (CFS 7–9)	48 (3.7)	29 (3.2)	19 (4.9)	0.120

CFS, clinical frailty score, *p-value accompanying comparisons in prevalence between genders

For further analysis frailty categories were condensed to represent non-frail (CFS 1–3), vulnerable (CFS 4), mildly (CFS 5), moderately (CFS 6) and severely frail (CFS 7–9). This is shown in detail in an additional file (see Additional file 1: Table S1).

### Prevalence of frailty according to gender, age and living status

#### Gender

Prevalence of frailty across categories did not significantly differ between males and females (Table 2). However, when all subjects were categorised as either robust (CFS 1–4) or frail (5–9), females were significantly more likely to be classed as frail [43% (*n* = 398) v 37% (*n* = 142), *p* = 0.033].

#### Age

**Fig. 2:**
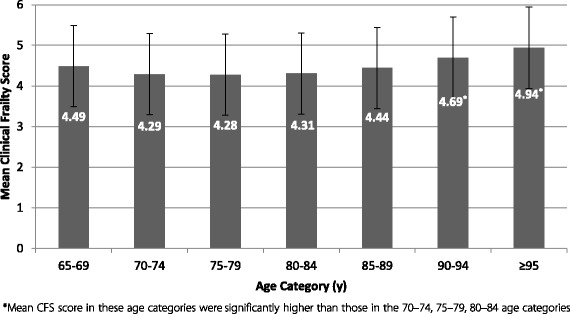
Mean and standard deviations of frailty scores (CFS) by age category (*n* = 1312)

The degree of frailty did not differ significantly across the younger categories aged 65–84 years (Fig. 1). In the oldest old, namely 90–94 and >95 years, the prevalence of moderate frailty was significantly higher relative to the younger age groups (21% and 34%, *p* < 0.05, *p* < 0.01 respectively) (Fig. 1). Consistent with the prevalence findings, an increase in mean frailty scores was observed from age 85 onwards (Fig. 2). Further analysis detected significantly higher mean CFS score within the oldest age groups only, with a small yet significant increase in frailty score in those 90–94 years compared with the younger categories 70–74 group (*p* = 0.049), 75–79 (*p* = 0.006), and 80–84 (*p* = 0.005). Similarly, CFS scores in the >95 category were also significantly elevated in comparison to younger groups (Table 3). Taken together the findings (Figs. 1, 2 and Table 3), suggest that age was not significantly associated with frailty status until later in life in this cohort of community dwelling elderly receiving home help.

**Table 3:** Post-hoc comparisons of differences in mean CFS scores between 90 and 94 & >95 groups and all age categories

	Age categories (years)						
	65–69	70–74	75–79	80–84	85–89	90–94	≥95
90–94	0.20	0.40*	0.41*	0.38*	0.26	-	−0.25
>95	0.45	0.65*	0.66*	0.63*	0.51	0.25	-

Mean differences shown. *difference is mean CFS score is significant at *p* < 0.05

#### Carer and living status

The highest prevalence of frailty, predictably, was among those cared for by others, namely by family excluding spouse (83.9%, *n* = 89) or by a spouse/partner (78.4%, *n* = 58) (Table 4). The majority of self-caring were classed as vulnerable according to the CFS (42.4%, *n* = 472) with over a further one-third [34.6%, *n* = 384)] of self-carers deemed frail; however only 1.2% (13) were severely or very severely frail, or terminally ill (CFS 7–9). Prevalence of moderate frailty – terminally ill (CFS 6–9) was significantly higher in the elderly being cared for by family (*p* < 0.001) and a spouse/partner (*p* < 0.001). Self-carers were more likely to be very fit – managing well (*p* < 0.001), vulnerable (*p* < 0.001) or mildly frail (*p* = 0.650), compared to those cared for by a spouse/partner however. Analysis of frailty according to living status (i.e. alone or with others) paralleled the principal care findings and are shown in an additional file (see Additional file 2: Table S2).

**Table 4:** Prevalence of frailty according to principal carer (*n* = 1292)

Clinical Frailty Scale, n(%)	Principal Carer				
	Self	Spouse/Partner	*p*-value*	Other Family	*p*-value**
Very fit – Managing Well (CFS 1–3)	256 (23.0)	2 (2.7)	<0.001	5 (4.7)	<0.001
Vulnerable (CFS 4)	472 (42.4)	14 (18.9)	<0.001	12(11.3)	<0.001
Mildly frail (CFS 5)	251 (22.6)	15 (20.3)	0.650	33 (31.1)	0.047
Moderately frail (CFS 6)	120 (10.8)	29 (39.2)	<0.001	37 (34.9)	<0.001
Severely frail – Terminally ill (CFS 7–9)	13 (1.2)	14 (18.9)	<0.001	19 (17.9)	<0.001

*CFS* clinical frailty scale

*p-value corresponding with comparisons between prevalence of frailty amongst self-caring and spouse/partner care group

**p-value corresponding with comparisons between prevalence of frailty amongst self-caring and 'other family' care group

The high prevalence of frailty in those cared for primarily by others was likely due to a significantly older, more dependent profile in these subgroups, as evidenced by a higher mean age when principal carer was family compared to self-caring (85.0, SD 7.98 v 81.9, SD 7.22, *p* < 0.001). Although there was no significant difference in age between those who were self-caring and older adults who were cared for by a spouse/partner (*p* = 0.129).

### Home help utilisation and frailty

**Fig. 3:**
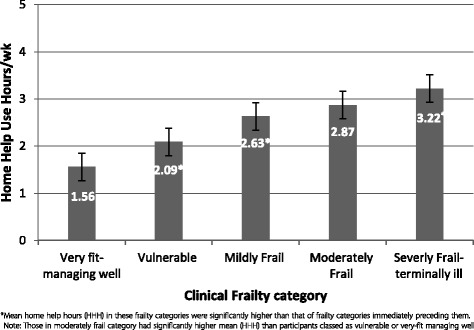
Home help utilisation across Clinical Frailty (CFS) categories

Home help utilisation increased statistically significantly according to the degree of frailty (Fig. 3 and Table 5) and positively correlated with frailty scores (*r* = 0.358, p = <0.001). The severely frail – terminally ill category (CFS 7–9) received the highest number of home help hours (mean 3.2, SD 1.58), two fold higher than the ‘very fit-managing-well’ category (CFS 1–3) (mean 1.6, SD 0.92). Similarly, those classed as moderately frail utilised significantly more home help hours than those classed as vulnerable [2.87 (SD 1.37) v 2.09 (SD 1.16), *p* < 0.05]. Interestingly, mean home help hours did not vary significantly between age categories as determined by one-way ANOVA (F (6,1302) = 1.613, *p* = 0.14), suggesting frailty status was a better indicator of home help in this cohort. The correlation between home help hours and age was weak and not statistically significant (*r* = 0.024, *p* = 0.393).

**Table 5:** Post-hoc comparisons of differences in mean home help hours across Clinical Frailty categories

	Vulnerable	Mildly Frail	Moderately Frail	Severely Frail – Terminally ill
Very fit- Managing well	−0.53*	−1.08*	−1.31*	−1.67*
Vulnerable	-	−0.55*	−0.78*	−1.14*
Mildly Frail		-	−0.24	−0.59*
Moderately Frail			-	−0.36*

Mean differences shown. *difference in means significant at *p* < 0.05

### Factors associated with frailty

Frailty, as determined by the CFS, significantly and positively correlated with dependency (Barthel Score) and with the number of home help hours received, non-self-caring, and communication difficulty (Table 6). The association with age, although significant, was weak, (rho = 0.1, *p* = 0.034) in agreement with earlier findings showing similar frailty status across chronological age categories up until the age of 90 years and above (Fig. 2, Table 3). Regression analysis confirmed the correlation results (Table 7) with Barthel score, home help hours, self-caring status and communication contributing significantly to the model, with an r^2^ value of 0.508.

**Table 6:** Factors associated with Frailty (Clinical Frailty Scale)

Variable	Spearman’s Correlation coefficient (r_s_)	*P* value
Age	0.101	0.034
Gender	−0.059	<0.001
Living Status (with others)	0.172	<0.001
Principal Carer Level (non-self)	0.371	<0.001
Home Help Hours	0.371	<0.001
Communication level	0.220	<0.001
Barthel Category^a^	0.609	<0.001

CFS 1–9 was used in this analysis. ^a^Barthel category: 1(independent) – 5 (maximum dependency)

**Table 7:** Multiple regression analysis model for frailty (Clinical frailty scale)

	B	se	Beta	*P* value
Constant	5.253	0.295		<0.001
Barthel Score	−0.223	0.009	−0.586	<0.001
Home help hours	0.123	0.017	0.157	<0.001
Principal care level	0.157	0.042	0.086	<0.001
Age	0.009	0.003	0.061	0.003
Communication level	0.088	0.039	0.049	0.023
			R squared	0.508

## Discussion

The growing ageing population [10], along with goals to promote ageing in place are increasing the demand for and use of government funded home help services in Ireland. There is limited published information on the health and social care profiles of home help users, including determinants of adverse outcomes, such as frailty, in this cohort. The aim of the present study was to identify and profile a cohort of older people in receipt of government funded low level home help on a regular basis, and to investigate the prevalence of frailty in this population.

The present study identified a unique population of 1312 urban community-dwelling older persons over 65 years of age in receipt of low level home help. We documented that the group was predominantly female, with a mean age of 82 years (SD 7.3). Subjects primarily lived alone, were self-caring, over half were widowed and the majority had no reported difficulties with communication and were largely rated as independent or low dependency. Based on the limited comparison data available, this demographic profile seems consistent with older adults in receipt of home care reported elsewhere [26].

The core aim was to determine frailty prevalence in a low-level home help population, using a simple practical measure, namely the Clinical Frailty Scale. Frailty was detected in 41.3% of older adults, with a further 38.4% being categorised as vulnerable. While published clinical frailty data in Ireland within any home help cohort is sparse, a recent Irish study by O’Caoimh et al. [31] reported frailty scores for 803 older adults living in the community or in sheltered housing in predominantly urban and suburban areas, with a mean age of 79.8 (SD 7.4) years. Participants had a comparable profile to our study in terms of gender ratios and Barthel Index score [31] and included subjects who may be considered more vulnerable, capturing only older persons in the community who were regularly monitored by a public health nurse, however only half of the group were in receipt of some form of home help (52%). Based on the Clinical Frailty Scale, O’Caoimh et al. reported frailty at 54.3% (mildly frail-terminally ill), higher than the prevalence of 41% in our present study, likely reflecting differences in the two study populations. A recent Canadian study by Campitelli et al. [14] in a similar cohort of urban community dwelling elderly (mean age 82.0) in receipt of formal home help, determined frailty by three different measures, namely the frailty index, modified frailty index and the CHESS frailty scale [32]. Prevalence varied from 19.5% - 44.1% depending on the methodology used. This large variation in prevalence due to lack of standardised procedures for detecting frailty in older populations is consistent with previous results reported [14]. Nonetheless, there is increasing emphasis on detecting frailty in the community, illustrated in the United Kingdom, for example, in the development of frailty care-pathways in primary care [33].

Additionally, we aimed to determine factors associated with frailty, measured by the Clinical Frailty Scale. From correlational analysis Activities of Daily Living (ADL) disability was the strongest associated factor, whilst being non-self caring, home help hours and communication level were moderately associated with frailty. Following regression analysis, Barthel Index, home help hours, principal care level, age and communication level remained significant determinants of frailty.

Elderly subjects presented with similar degrees of frailty up to 89 years, with age only weakly correlating with frailty. Similar results were presented from the O’Caoimh study, in which frailty was only weakly associated with age [31]. These findings would suggest that chronological age alone, particularly in high risk cohorts, is not a significant determinant of frailty status, and factors such as clinical condition and cognitive impairment may have a more important role to play in the trajectory from robust to frail. Whilst limited health-related data were available to us, this has previously been demonstrated elsewhere [31, 34]. This observation is further strengthened by the association seen between communication level, likely reflecting cognition or an acute insult such as stroke, and frailty in the present study. Furthermore, the finding that prevalence of frailty, while increasing marginally but only increased significantly after the age of 90 years, presents a potential opportunity for prevention strategies in order to address certain modifiable factors and to slow or halt the progression from prefrail or vulnerable to frail. There are a number of proposed interventions which target aspects of frailty or physical frailty, including exercise and nutrition [35] as well as technology based approaches. Recent results from intervention trials are promising and affirm the role of multi-modal approaches, including exercise and nutrition, in managing and preventing frailty in the elderly [13, 36–39].

Home help hours were significantly associated with frailty following regression analysis, however home care utilisation did not significantly correlate with chronological age which is in agreement with previous Irish data [31]. Frailty has consistently been shown to be a significant contributor to increased utilisation of home care services in community dwelling elderly [31, 40], independent of potential confounders including age and gender [41]. In a recent Irish population-based study, factors reported as predictive for home help utilisation included living alone, older age and Independent Acitivities of Daily Living (IADL) difficulty [12]. However, the study did not include a measure of frailty, and authors noted that due to this the size of the effect of age on home care utilisation may have been overestimated [12].

Other associated factors included ADL disability (Barthel score), gender and principal care level. The low level of dependency in ADLs corresponds well with results from a previous study involving a similar cohort of elderly in the community availing of PHN services [26]. Dependency or ADL disability and its relationship with frailty, particularly physical frailty, is well documented in the literature [20, 25, 42, 43]. Whilst beyond the scope of the present study, there is an abundance of evidence showing that frailty is a significant risk factor for other adverse events including falls, fractures [42], hospitalisation [20, 41], institutionalisation into long term care [44, 45] and death [16, 21, 45]. Thus, frailty may be applicable to predict the trajectory of elderly home help users in the community. O’Caoimh et al. showed those classed as frail according to CFS had the highest prevalence of perceived one-year risk of adverse outcomes. According to recent data by O’Caoimhe [24], fit-managing well conferred a 18% maximum risk of hospitalisation within one year, vulnerable a 25.1% and frail a 48.3% of hospitalisation. Interestingly, further work by O’Caoimhe [46] highlighted that CFS was a reasonable predictor of mortality, and institutionalisation but none of the measures were reliable predictors of hospitalisation. The clear link between frailty and adverse outcomes further strengthens the need for targeted intervention programmes, particularly in an higher risk cohort of elderly, to support successful ageing and slow the increasing demand for health and social care services amongst older people living at home.

The association between female gender and frailty did not remain significant following regression analysis. Whilst more males were classed as very fit – managing well in comparison to females (*p* = 0.060), they were more likely to be classified in the severely frail – terminally ill category (*p* = 0.120). This finding has been mirrored in studies involving more vulnerable populations which show inconsistent association with gender [32], as well as an increased likelihood of males falling into the most severe frailty categories [47].

This study represents a large population (*n* = 1312) with limited published data, especially regarding frailty and factors that may predict adverse outcomes. A number of limitations must be mentioned, for example the reliance on clinical data means several important variables were unavailable including, socio-economic status, lifestyle factors, health status and co-morbidities, cognitive assessment, and duration of home help service usage. We applied the CFS as a simple practical indicator of frailty, nonetheless there are known limitations associated with subjective scores of frailty reliant on clinical judgement, with multiple and more comprehensive frailty tools available [21, 33]. Furthermore, an assessment of inter-rater reliability was not completed in the present study and thus variation between nurses in the use and interpretation of the CFS assessment cannot be ruled out. Nevertheless, all PHNs had extensive experience in community nursing and care of the elderly, and thus their clinical judgement along with written criteria and guidance provided on assessment forms would likely reduce heterogeneity in interpretation of clinical and functional symptoms and thus scoring on the CFS. Future studies could aim to follow up participants and capture the frail ‘phenotype’ by objective measures such as handgrip strength and gait speed [20]. To note, we studied frailty among those with low level home help (<5 h per week) as this may identify earlier opportunity for intervention and prevention strategies; greater than >5 h of home help, termed ‘home care packages’, were not included in the analysis, so all levels of home help service are not represented.

## Conclusion

In conclusion, we identified a high prevalence of frailty (41.5%) in this cohort which was associated with higher home help utilisation, despite including only those receiving low level home help (<5 h/week). Determinants of frailty included greater dependency (Barthel score), higher home help hours, non-self-caring and communication difficulty. Interestingly**,** frailty was not strongly associated with chronological age, until after 90 years. These findings highlight opportunities for earlier intervention strategies targeted at frailty, that promote ageing in place in populations already in contact with the health service, through the regular use of home help services. Inclusion of frailty measures, particularly objective measures of physical frailty, across community services for older people including home help services, may be useful for the provision of tailored individual support, such as reablement programmes, to those most at risk. Research has indicated that time limited home care in the form of reablement can lead to improvements in functional abilities and a reduction in the need for on-going home care support [48–50].Incorporating frailty measures into community services may also be beneficial for longer term health and social care service planning.

## Additional files

Additional file 1: Table S1. Prevalence of frailty categories (condensed) overall and by age categories. (XLSX 10 kb)

Additional file 2: Table S2. Prevalence of frailty according to living status. (XLSX 9 kb)

## Abbreviations

ADL: Activities of Daily Living
CFS: Clinical Frailty Scale
CSAR: Common Summary Assessment Report
HSE: Health Service Executive
PHN: Public health nurse
